# Discussion on Assessment and Treatment of Cognitive Impairment and New Developments of Cognitive Remediation in Schizophrenia

**DOI:** 10.1192/j.eurpsy.2022.165

**Published:** 2022-09-01

**Authors:** T. Wykes

**Affiliations:** King’s College London, Institute Of Psychiatry, Psychology And Neuroscience, London, United Kingdom

**Keywords:** schizophrenia, cognitive remediation, psychological therapy

## Abstract

Cognitive impairment is conceptualised in different ways and the discussion will highlight differences and similarities. The importance of these cognitive impairments for choosing specific or generic cognitive remediation therapies will be highlighted as well as the need to consider social cognition and metacognition as potentially more important ingredients for improving recovery.

**Disclosure:**

No significant relationships.

